# Treasurer’s Report for Financial Year (FY) 2020

**DOI:** 10.1093/molbev/msab320

**Published:** 2021-11-22

*Prepared by John P. McCutcheon, SMBE Treasurer, Audited by Jeffrey L. Thorne*

The society’s assets and liabilities as of December 31, 2020, are as follows:

**Table:** 

Assets	
Cash account	$1,839,852.05
CDs	$2,127,645.04
Prepaid expenses	$118,000.00
Accounts receivable	$248,950.09
Total	$4,334,447.18
Liabilities	
Accounts payable	$123,745.40
Deferred membership	$23,732.56
Deferred journal revenue	$642,037.00
Total	$789,514.96
Net assets	$3,544,932.22

This report covers the 12 months from January 1, 2020 to December 31, 2020. The Society continues to be in very good financial health. For 2020, the net revenue collected by the Society was $650,080.41, with the main sources of revenue being our two journals, *Molecular Biology and Evolution* (*MBE*) and *Genome Biology and Evolution* (*GBE*).

In a normal year, the majority of the Society’s expenses would have been dedicated to the annual meeting (and these expenses would have significantly lowered the net revenue for the year). However, the annual meeting was canceled in 2020 due to the COVID pandemic. This cancelation meant that travel expenses for SMBE Council and *MBE* and *GBE* editors were trivial compared with a normal year. Additionally, no Carer Awards or student travel awards were given. The $100,773.21 in expenses related to the annual meeting in this 2020 report were due to several payments for refunds related to the canceled 2020 meeting. In 2020, the Society still awarded the Walter Fitch Prize, the Best Student Paper Award, and various other awards.

For several previous years, the fiscal year for *MBE* and *GBE* did not align with the calendar year. In concordance with decisions made by the SMBE Council at the 2019 annual meeting, this Treasurer’s report directly reflects the journal revenues of 2020, now perfectly matched to the calendar year. This alignment of calendars greatly facilitates the reporting of yearly journal revenues by eliminating the need to estimate several months of future revenues, which would sometimes result in revenue adjustments in the previous year’s reports.

This 2020 report will also be the last where some fraction of *MBE* revenue comes from a subscription model (45% in FY20). It will be of great interest how the change to 100% open access (OA) will affect the revenue for *MBE*. *GBE* continues to function as a full OA journal, as it has since its inception.

Society’s Revenues and Expenses for FY2020

**Table:** 

Revenues	
Membership dues	$16,365.86
MBE FY2020 revenue	$674,800.04
Member subscription revenue	$2,354.61
GBE FY2020 revenue	$323,887.00
Unrestricted donation	$600.55
Interest income	$43,334.26
*Total* *revenues*	*$1,061,342.32*
Expenses	
Senior editor travel	$0.00
Associate editors travel	$0.00
Past/present editors-in-chief and journal staff travel	$0.00
Council travel	$2,010.42
Fitch symposium travel	$0.00
Undergraduate diversity travel	$0.00
Young investigator travel	$0.00
*Total* *travel*	*$2,010.42*
Miscellaneous prize and career awards	$14,500
Carer awards	$0.00
*Total* *prize and* *awards*	*$14,500*
Operating expenses	$62,828.52
Bank charges	$929.92
Tax preparation fees	$4,577.50
Legal fees	$7,395.97
*Total* *operations*	*$75,731.91*
*MBE* editing	$91,000.00
*MBE* highlight honoraria	$0.00
*GBE* editing	$100,000.00
*GBE* highlight honoraria	$22,551.00
*Total* *editorial*	*$213,551.00*
Annual SMBE meeting	$100,773.21
Other meeting/workshop sponsorships	$4,695.37
*Total* *meetings*	*$105,468.58*
*Total* *expenses*	*$411,261.91*
*Change in* *net* *assets*	*$650,080.41*

